# A machine learning model of lamina propria fibrosis in eosinophilic esophagitis for prediction of fibrostenotic disease

**DOI:** 10.1016/j.jpi.2025.100538

**Published:** 2025-12-22

**Authors:** Priyadharshini Sivasubramaniam, Abdelrahman Shabaan, Rofyda Elhalaby, Bashar Hasan, Ameya A. Patil, Saadiya Nazli, Adilson DaCosta, Byoung Uk Park, Lindsey Smith, Taofic Mounajjed, Stephen M. Lagana, Chamil Codipilly, Puanani Hopson, Imad Absah, Christopher P. Hartley, Rondell P. Graham, Roger K. Moreira

**Affiliations:** a Pathology And Laboratory Medicine, Medical College of Wisconsin, Milwaukee, WI, USA; b Department of Laboratory Medicine and Pathology, Mayo Clinic, Rochester, MN, USA; c Department of Pathology, University of Minnesota, Minneapolis, MN, USA; d MySME, LLC; Mayo Clinic, Rochester, MN, USA; e Department of Pathology and Cell Biology, Columbia University Irving Medical Center, New York, NY, USA; f University of Virginia, Charlottesville, VA, USA

**Keywords:** Eosinophilic esophagitis, Lamina propria fibrosis, Fibrosis, Stricture, Stenosis, Machine learning, Artificial intelligence, AI, Digital pathology, Histological assessment, Whole-slide imaging, Predictive modeling, Esophagus

## Abstract

**Background:**

Eosinophilic esophagitis (EoE) is a chronic immune-mediated disease that can progress to fibrostenotic complications. Lamina propria fibrosis (LPF) plays a critical role in this progression but is difficult to assess reliably in routine biopsies. We aimed to develop and validate an artificial intelligence (AI) model to quantify LPF on hematoxylin and eosin (H&E)-stained slides and to evaluate its ability to predict fibrostenotic disease.

**Methods:**

We used a cloud-based platform (Aiforia Inc., Cambridge, MA, USA) to train a supervised AI model to recognize several histological features of EoE, including LPF. Our validation cohort consisted of 213 esophageal biopsy whole-slide images, including 100 adult and 113 pediatric samples with mucosal eosinophilia, which were prospectively evaluated in our anatomic pathology service between 2020 and 2021 using a standardized histological scoring system. AI-based LPF scores were correlated with the development of fibrostenotic disease on subsequent endoscopies after a median follow-up time of 31.4 months.

**Results:**

The AI fibrosis score correlated with pathologist-determined LPF (Spearman's *R*s = 0.64–0.69, *p* < 0.0001) and outperformed pathologists' assessments in predicting fibrostenotic outcomes. Higher AI fibrosis scores were associated with the development of rings, strictures, and need for dilatation on follow-up (*p* < 0.01), including in cases deemed histologically inadequate by pathologists and in the subgroup without prior strictures. In a Cox Proportional-Hazards model, the AI fibrosis score was an independent predictor of strictures (C-index = 0.73, *p* = 0.004). Importantly, meaningful predictions were achievable with smaller amounts of lamina propria than traditionally deemed sufficient.

**Conclusion:**

This study demonstrates that AI-based quantification of LPF on routine H&E slides provides an objective and clinically meaningful assessment of fibrosis in EoE. The AI fibrosis score predicts fibrostenotic disease more consistently than conventional pathology evaluation and may improve risk stratification even in limited biopsy samples. Integration of digital pathology tools may enhance histological assessment of fibrosis in EoE and support clinical decision-making.

## Introduction

Eosinophilic esophagitis (EoE) is a chronic immune-mediated disease of the esophagus affecting both children and adults. Pathologically, EoE is characterized by a combination of eosinophilic inflammation, features of squamous epithelial injury, and fibrosis, the latter being an important contributor to disease chronicity and symptom persistence.^1^^,^^2^ Whereas eosinophilic infiltration is central to the pathogenesis of EoE, the role of fibrosis, particularly in the lamina propria (LP), has gained increasing attention due to its role in esophageal dysfunction and stricture formation.^1^^,^^3^ Although the pathogenesis and prevalence of tissue fibrosis in EoE are not well characterized, the disease is currently thought of as a predominantly inflammatory disorder early in its course, progressing to a mixed inflammatory/fibrostenotic disease after several years, and finally, to a predominantly fibrostenotic disease after a decade or more of untreated disease.^3^ Based on data from murine models and from sporadic case reports of surgical specimens with esophageal fibrosis, EoE is known to involve all layers of the esophagus, including the muscularis propria.^4^, ^5^, ^6^ However, the LP is the only subepithelial layer that is routinely sampled, albeit inconsistently, for histopathological examination with current endoscopic biopsy techniques, hence its relevance in this context.

Traditional histopathological assessment has been limited in its ability to accurately identify and quantify lamina propria fibrosis (LPF) in EoE. A precise definition of LPF is difficult to establish (various definitions in the literature summarized by Thaker et al.^7^) and its evaluation relies on pathologists recognizing subtle changes in subepithelial connective tissue, which can be affected by disease severity, procedure-related factors (e.g., crush artifact), and variability of histological sectioning and staining. As a result, interobserver agreement in the assessment of LPF has been relatively low, with reported interclass correlation coefficient of 0.58 and 0.61 for LPF grade and stage,^8^ respectively, in the adult population and agreement in only 57% of the samples (Krippendorff's α = 0.34) in children.^7^ In spite of these challenges, pathologists agree that the LP collagenous matrix undergoes changes in EoE that are detectable by light microscopy—and that the delicate, thin, organized collagen bundles in the normal LP progressively become thicker (aggregating in wider bundles), denser, coarser, and more hyalinized.

Assessment of adequacy for LPF evaluation presents its own set of challenges and is generally based on the subjective impression of “sufficient LP tissue” by pathologists, often with no objective criteria (such as a specific area or number of high-power fields) stipulated. When specified, criteria are often arbitrary (a minimal area of 75 × 150 μm in the work of Thacker et al.,^7^ or any amount of LP excluding artifacts in Wang et al.^9^) rather than based on their correlation with meaningful variables or predictive ability for specified outcomes.

The application of novel technologies, including digital pathology tools, has the potential to significantly enhance the value of histological assessment in this context by harnessing our existing histopathological knowledge of EoE-related LPF and instantiating it into machine learning algorithms. In addition, the current capabilities of AI models could help us mitigate the problem of reproducibility, establish more precise adequacy criteria by means of accurate measurement, and produce results that are both automated and scalable. In this study, we evaluated the utility of a machine learning model of histopathological assessment and quantification of LP and LPF in endoscopic biopsy samples from patients with esophageal eosinophilia.

## Materials and methods

### Study population

We analyzed a previously characterized cohort of 213 esophageal biopsy slides (100 adult and 113 pediatric samples) from 180 patients (92 adults, 88 children or adolescents) evaluated between 2020 and 2021 at a single large academic pathology practice.^10^^,^^11^ Consecutive adult and pediatric cases were selected from a prospectively maintained database of known or suspected EoE cases, as well as patients incidentally found to have intraepithelial eosinophils of any degree, with a standardized eosinophilic esophagitis histologic scoring system (EoEHSS)^12^ evaluation by the staff GI pathologist reviewing the case at the time of the original biopsy report. For the purpose of this study, patients were considered to have EoE if they were either labeled as such on medical records/clinical notes based on previous records or if they had >15 eosinophils per HPF on a biopsy sample (as assessed by the original pathologists, not AI model) in the absence of non-EoE conditions associated with significant eosinophilia, as per consensus guidelines.^13^

### Histological analysis

The original evaluation of each case was performed by a staff pathologist in the gastrointestinal pathology working group at our institution (12 pathologists in total, referred to as “original pathologist”, or OP). Peak eosinophil count (PEC) was provided and an EoEHSS synoptic report, which included eosinophilic inflammation (grade and stage), basal zone hyperplasia (grade and stage), dilated intercellular spaces (DIS) (grade and stage), LP fibrosis (grade and stage, or no score if the sample was inadequate for LPF evaluation), eosinophilic abscesses (present/absent), surface layering (present/absent), and total EoEHSS score was completed at the time of original diagnostic report. All cases were subsequently re-scored by a central pathologist (CP) (R.K.M.), for EoEHSS features, PEC, presence of any LP, and subjective assessment of LP adequacy. Active EoE was defined for the purposes of this study as PEC > 15 by either the OP or CP.

### Artificial intelligence model development

A supervised artificial intelligence (AI) model was developed using a cloud-based deep learning platform (Aiforia Inc., Cambridge, MA, USA). The training set consisted of 99 digitized whole-slide images (WSIs) from esophageal biopsies from four different institutions/histology labs in the USA, including pediatric and adult samples, and spanning the full spectrum of LP histological appearance, from normal (non-fibrotic) pattern to dense and diffuse fibrosis. The model employed a hierarchical structure of convolutional neural networks (CNNs). The parent layers of the model were designed to identify regions with the following child layer structure:

Lamina propria

1. Fibrotic pattern
  a. Collagen area
  b. Non-collagen area (including blood vessels, areas of neovascularization, and inflammatory infiltrate)
2. Non fibrotic pattern
  a. Collagen area
  b. Non-collagen area (including blood vessels, areas of neovascularization, and inflammatory infiltrate).

The CNNs were trained using a supervised approach, where annotated features were iteratively provided as inputs to improve accuracy. All WSIs were scanned at 40× magnification using the Aperio ScanScope AT Turbo brightfield instrument (Leica Biosystems) at 0.25 μm per pixel resolution. A subset of training cases was also scanned with two different scanners (Aperio GT-450 and AT3). Digital images were then uploaded to the Aiforia platform for AI-based analysis.

**Fig. 1:**
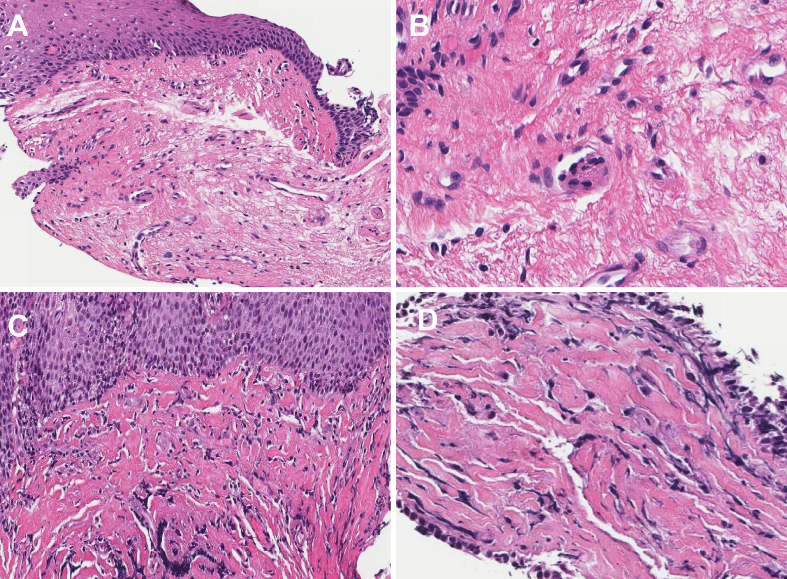
(A, B) Hematoxylin and eosin stains showing normal (non-fibrotic) appearance of the esophageal lamina propria. (C, D) Fibrotic lamina propria, featuring hyalinized, thickened collagen bundles in the setting of eosinophilic esophagitis.

**Fig. 2:**
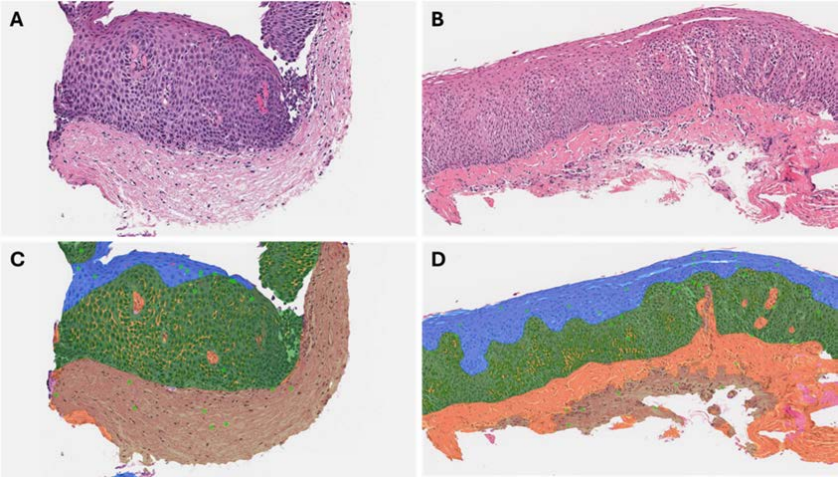
AI model recognition of LP and LPF in esophageal biopsies. Original hematoxylin and eosin stains showing biopsy samples from EoE patients without LPF (A) and with LPF (B). Notice the delicate, thin, normal collagen fibers in A in contrast with the thick, hyalinized collagen bundles in B. AI model semantic segmentation of the same samples, featuring a predominantly non-fibrotic pattern in the first case (shown in beige, C), compared to the predominantly fibrotic pattern in the second case (shown in orange, D). Epithelial segmentation showing basal layer (dark green) mature squamous epithelium (blue), dilated intercellular spaces (yellow) and eosinophils (light green dots). Abbreviations: AI, artificial intelligence; EoE, eosinophilic esophagitis; LP, lamina propria; LPF, lamina propria fibrosis. (For interpretation of the references to color in this figure legend, the reader is referred to the web version of this article.)

Pathology research fellows (P.S. and R.E.) were trained to recognize and classify LP and LPF and annotated these slides to identify and delineate the histological features of interest. Normal LP collagen was characterized by thin whisps of generally loose collagen, whereas the collagen in fibrotic LP appears denser, with thick, hyalinized bundles of collagen (Fig. 1), as per criteria suggested by Warmer et al.^8^ A subspecialized gastrointestinal pathologist then verified all annotations and made adjustments when necessary. The model employed semantic segmentation for training and analysis, with a total annotated surface area of 57.077 mm^2^ and 15,000 training iterations (advanced parameters are presented on Supplemental Table 1). This included 2.377 mm^2^ of fibrotic area and 7.291 mm^2^ of non-fibrotic area within the LP, as well as 0.717 mm^2^ of collagen area and 0.403 mm^2^ of non-collagen area. The Aiforia platform's internal validation tool was employed to evaluate the model's performance on features identified during training. Model performance was assessed by comparing its segmentation of LP and fibrotic regions with pathologist-determined ground-truth annotations in 99 validation WSIs (from 99 patients) at our institution. The LPF AI model was inserted within a comprehensive pre-existing AI model for other histological features of EoE, including eosinophils, lymphocytes, basal layer hyperplasia, DIS, and eosinophilic abscesses, as previously described by our group (Fig. 2).^10^^,^^11^

### Clinical and endoscopic data

Demographic, clinical, and endoscopic data, including fibrosis-related endoscopic findings (as part of the endoscopic reference score, EREFS),^14^ such as esophageal rings, strictures, strictures requiring dilatation, and episodes of food impaction (defined as an episode requiring endoscopic intervention), were collected from medical records up to February 2025. Data on all endoscopic examinations performed during or before the study period were recorded. Disease duration was established based on the date of initial diagnosis of EoE (as reported in the patients' records) to the date of study endoscopy/biopsy (in years). Institutional review board approval was obtained before the initiation of the study, and patient confidentiality was maintained in accordance with institutional policies.

### AI model metrics and performance evaluation

The performance of the AI model was evaluated using our platform's validation tool. The reported metrics included precision, sensitivity, and F1 score (Table 1). These metrics were calculated for each feature detected by the model, including LP area, fibrotic area, and non-fibrotic area. Because each slide contained a variable number of sections (levels) of the paraffin tissue block, the LP area value used in all calculations was the raw LP area recognized by the AI model (i.e., over the entire WSI, including in all histological levels) divided by the number of histological levels in the corresponding slide (average of 3.4 levels per slide).

**Table 1:** AI model performance on validation against pathologists' manual annotations.

Features	Precision (%)	Sensitivity (%)	F1 score (%)
Lamina propria	98.50	95.73	97.09
Fibrosis	99.08	99.05	99.07
Non-fibrosis	99.99	95.42	97.65

Basal zone	96.8	85.6	90.9
Mature epithelium	98.4	95.7	97.0
DIS	60.8	80.7	69.3

Eosinophils	98.7	95.7	97.1
Lymphocytes	98.3	88.4	93.1
Squamous cells	98.8	96.8	97.8

**Fig. 3:**
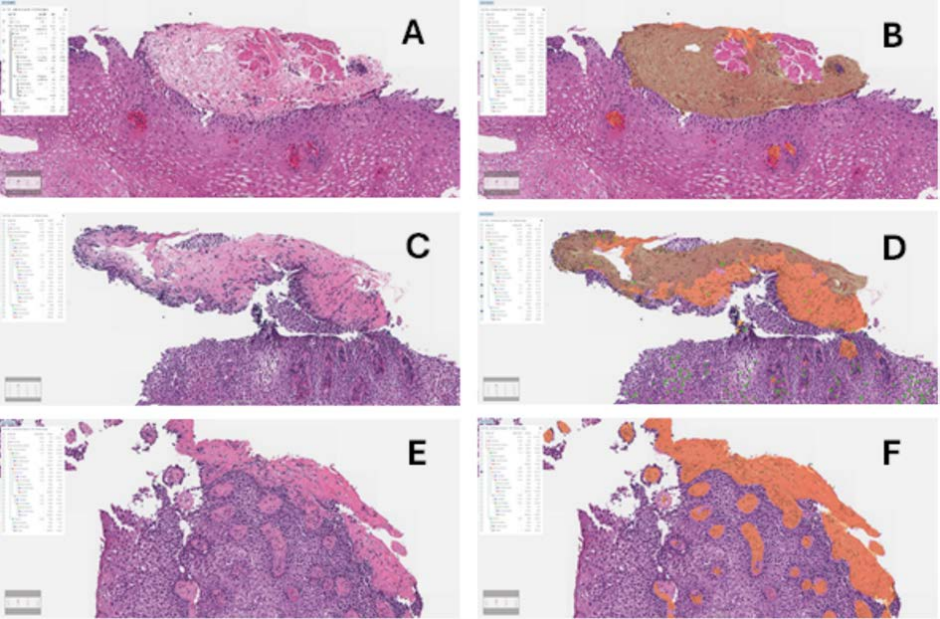
Artificial intelligence model of lamina propria fibrosis (AI-LPF). (A, B) Normal lamina propria (LP) highlighted in beige. Notice that the fragment of muscularis mucosa is excluded by the model. (C, D) Partially fibrotic LP with fibrotic area in orange and non-fibrotic area in beige. (E, F) Diffusely fibrotic LP highlighted in orange.

The AI model quantified the area of LP and, within this layer, the area of fibrotic and non-fibrotic patterns (Fig. 3). The model then calculated both the absolute fibrotic area (mm^2^) and the relative fibrotic area (percentage of LP occupied by a fibrotic pattern, or % fibrosis). The final AI model fibrosis score (used in all statistical analyses) was calculated by multiplying the fibrosis area by the fibrosis% (fibrosis area/total LP area). For example, if fibrosis area = 0.5 mm^2^ and fibrosis% is 50%, then, the AI fibrosis score would be 0.5 × 50 = 25.

### Clinical utility survey among gastroenterologists

A brief, structured survey assessing the perceived clinical utility of LPF assessment in EoE was distributed to adult and pediatric gastroenterologists at our institution. The full survey content and responses are provided in Supplemental Table 2.

### Statistical analysis

All statistical analyses were performed using MedCalc Statistical Software version 20.0 (MedCalc Software Ltd., Ostend, Belgium). Spearman correlation coefficients were calculated to assess the relationship between AI- and pathologist-derived fibrosis scores. Multiple regression models were used to evaluate the ability of AI-derived LPF measurements to predict endoscopic findings on follow-up, including other EoE-related features (PEC by the OP and CP, and total EoEHSS by the OP and CP) in a stepwise fashion. Interobserver agreement between pathologists was assessed by unweighted (or with linear weight, if appropriate) Cohen's Kappa analysis. Shapiro–Wilk test was used to determine normality in data distribution. Whitney *U* test was used to compare differences in median AI fibrosis score between groups (two-tailed probability). Kaplan–Meier analysis was performed for specific outcomes, including the development of mucosal rings, strictures, and strictures requiring balloon dilatation procedure endoscopically, using optimal cutoff points of the AI model fibrosis score calculated by receiver operating characteristic (ROC) analysis. Statistical significance was set at *p* < 0.05. All LP adequacy data were analyzed on a per slide basis, whereas correlation with endoscopic findings was analyzed on a per patient basis.

## Results

### Demographic and clinical features

The demographic, clinical, and pathological baseline characteristics of our study population are described in Table 2. One hundred and sixteen (116) of 180 (64.4%) patients had at least one follow-up endoscopy, with a median follow-up time of 31.4 (range: 1.8–60.7) months. Age and disease duration were the most significant clinical features in prediction of fibrostenotic disease. In a statistical model that also included PEC, total EoEHSS, and AI model LPF score (Stepwise Cox Proportional-Hazards), patient age and disease duration were independently predictive of rings (*p* = 0.003) and strictures (*p* = 0.004) on subsequent EGDs and disease duration was independently predictive of strictures requiring dilatation (*p* = 0.02). Sex, history of atopy, and ethnicity were not predictive of rings, strictures, or strictures requiring dilation on follow-up endoscopies in the entire cohort or subgroup analysis.

**Table 2:** Demographic, clinical, and pathological baseline characteristics of our study population. Abbreviations: PPI, proton-pump inhibitor; EoE, eosinophilic esophagitis; PEC, peak eosinophilic count; EI, eosinophilic inflammation; BZH, basal zone hyperplasia; DIS, dilated intercellular spaces; LPF, lamina propria fibrosis; LP, lamina propria; EoEHSS, eosinophilic esophagitis histologic severity score; EREFS, endoscopic reference score; EGD, esophagogastroduodenoscopy; FSD, fibrostenotic disease. * Numbers are unique patient-based; ** Numbers are sample-based.

	All patients, *n* = 180	Adult, *n* = 92	Pediatric, *n* = 88
Clinical/demographic: *			
Age (range)	26.9 (1.2–75.0)	43.0 (18–75.0)	12.4 (1.2–17.9)
Gender			
Male	112 (62.2%)	50 (54.3%)	62 (70.5%)
Female	68 (37.8%)	42 (45.6%)	26 (29.5%)
Allergic disease	113 (62.7%)	50 (54.3%)	63 (71.5%)
Pre-biopsy treatment			
Topical steroids	86 (47.7%)	36 (39.1%)	50 (56.8%)
PPI	60 (33.3%)	25 (27.1%)	35 (39.7%)
Dietary restriction	103 (57.2%)	48 (52.1%)	55 (62.5%)
Diagnosis of EoE	166 (92.2%)	81 (88.0%)	85 (96.5%)
Disease duration EoE patients, years (SD)	3.1 (10.0)	3.9 (13.7)	2.3 (3.2)

Histological features, avg. (SD): **			
PEC	42.4 (31.7)	40.7 (31.9)	45.2 (30.7)
EI grade	2.0 (0.9)	2.0 (1.0)	2.2 (0.8)
EI stage	1.7 (1.1)	1.6 (1.1)	1.8 (1.1)
BZH grade	1.8 (1.0)	1.8 (1.0)	1.9 (0.9)
BZH stage	2.0 (1.1)	1.9 (1.1)	2.1 (1.0)
DIS grade	2.0 (1.1)	1.9 (1.2)	2.1 (1.1)
DIS stage	1.6 (1.1)	1.5 (1.0)	1.7 (1.0)
LPF grade	1.4 (1.1)	1.3 (1.1)	1.6 (1.0)
LPF stage	1.3 (1.1)	1.2 (1.1)	1.5 (1.1)
Total EoEHSS	12.4 (6.4)	12.1 (6.7)	13.2 (5.9)

EREFS (index endoscopy).			
Total EREFS	2.4 (1.7)	2.4 (1.9)	2.5 (1.7)

Strictures before index EGD*	7 (3.8)	7 (3.8)	0 (0.0)
Strictures on index EGD*	23 (12.7)	20 (11.1)	3 (1.6)
Strictures on follow-up EGD*	24 (13.3)	20 (11.1)	4 (2.2)
FSD requiring dilatation on follow-up EGD*	23 (12.7)	20 (11.1)	3 (1.6)

### Clinical utility survey among gastroenterologists

The survey was distributed to 17 Mayo Clinic gastroenterologists (9 adult and 8 pediatric), with responses received from 13 clinicians (6 adult and 7 pediatric). Respondents showed a similar distribution of early career and more experienced gastroenterologists, with representation across all years-in-practice categories. The number of EoE-related endoscopic biopsy procedures performed per month varied widely (median, 15; range, 5–30). LPF was most commonly reported to have a weak to moderate influence on disease severity assessment beyond PEC, although most respondents considered fibrosis information to be at least moderately clinically useful. Perceived correlation between LPF and endoscopic features, such as the pull or tug sign, was generally rated as moderate, and most gastroenterologists felt that LPF may reflect deeper wall involvement, but with uncertainty. Whereas few respondents felt that LPF clearly predicted future fibrostenotic complications, the majority indicated that such information could be clinically actionable if supported by additional evidence, and most reported that a validated AI-based fibrosis score would influence management, most commonly by increasing monitoring frequency and/or escalating therapy. The additional response details are presented in Supplemental Table 2.

### Pathologists' assessment of LPF and correlation with fibrostenotic disease

The OP and CP diagnosed LPF (of any degree) in approximately one-third of all samples (34.3% and 34.7%, respectively). The interobserver agreement on fibrosis (any vs none) between OP and CP was moderate (Kappa = 0.42) for all samples. In the adult subgroup, the OP and CP diagnosed LPF in 39.4% and 36.4%, respectively, with an interobserver agreement of 0.50. In the pediatric population, the OP and CP diagnosed LPF in 29.8% and 33.3%, respectively, with an interobserver agreement of 0.35 (Table 3).

**Table 3:** Lamina propria adequacy and lamina propria fibrosis rates, and interobserver agreement between the original and central pathologist in different subgroups. Numbers reflect analyses on a per sample basis. Abbreviations: *n*, number of cases; LP, lamina propria; EoE, eosinophilic esophagitis; LPF, lamina propria fibrosis.

Category	*n*	Original pathologist	Central pathologist	Cohen's Kappa
LP adequacy (all)	213	106 (49.7%)	101 (47.4%)	0.38 (0.27–0.51)
LP adequacy (adult)	99	67 (66.6%)	48 (48.5%)	0.40 (0.22–0.58)
LP adequacy (pediatric)	113	40 (35.0%)	52 (46.0%)	0.37 (0.20–0.55)
LP adequacy EoE	197	97 (49.2%)	98 (49.7%)	0.42 (0.29–0.55)
LP adequacy active EoE	176	85 (48.3%)	90 (51.1%)	0.44 (0.31–0.58)
LP adequacy inactive EoE	37	21 (56.8%)	11 (29.7%)	0.18 (−0.12–0.48)
LPF (all samples)	213	73 (34.3%)	74 (34.7%)	0.42 (0.30–0.56)
LPF (adult)	99	39 (39.4%)	36 (36.4%)	0.50 (0.33–0.68)
LPF (pediatric)	113	33 (29.2%)	38 (33.6%)	0.35 (0.16–0.54)
LPF EoE	197	73 (37.1%)	73 (37.1%)	0.41 (0.28–0.55)
LPF active EoE	176	70 (39.8%)	70 (39.8%)	0.40 (0.27–0.55)
LPF inactive EoE	37	3 (8.1%)	4 (10.8%)	0.21 (−0.43–0.85)

Assessment of fibrosis by the OP and CP correlated with presence of endoscopic fibrosis features in some subgroups, particularly with fibrostenotic disease requiring dilatation on follow-up EGD, including in patients without prior history of strictures. However, the correlation between pathologists' LPF assessment and fibrostenotic disease was not consistent between OP and CP or across outcomes (see Supplemental Table 3). Neither OP nor CP readings correlated with future episodes of food impaction in the study follow-up period.

### AI model measurement of LPF—correlation with pathologists and prediction of fibrostenotic disease

In the OP evaluation, adequate samples with LPF had a significantly higher AI fibrosis score compared to adequate samples without LPF (34.1 vs 6.6, respectively, for cases with and without LPF, *p* < 0.0001). Equivalent results were seen with interpretations by the CP (47.9 vs 6.7, respectively, for cases with and without LPF, *p* < 0.0001) (Supplemental Table 4). ROC analysis for the presence of fibrosis showed an AUC of 0.80 at an AI fibrosis score > 19.3 and an AUC of 0.90 at an AI fibrosis score > 18.6 (*p* < 0.001) for the OP and CP, respectively. The AI model LPF score also correlated with the OP composite LPF score (LPF grade + LPF stage) (*R*s = 0.69, *p* < 0.0001) and with the CP composite LPF score (*R*s = 0.64, *p* < 0.0001). The correlation of the AI LPF score with various EoEHSS and AI model parameters is presented in Supplemental Table 5.

The AI model LPF score was superior to pathologists' assessment of LPF in correlating with fibrosis-related findings on both index and follow-up endoscopies. For the index endoscopy, AI LPF score correlated with the presence of rings in the adult subgroup and in subgroups with different minimal LP cutoffs, starting at 0.05 mm^2^ (Table 4A). On follow-up endoscopy, AI LPF scores were predictive of the development of rings in all subgroups, except in the pediatric population. AI LPF scores were also predictive of strictures and strictures requiring dilatation in all cases, in most subgroups, and at different minimal amount of LP cutoffs (Table 4B). ROC analysis showed an AUC of 0.67 (*p* = 0.008) for the prediction of strictures on follow-up EDGs for the entire study cohort, at an AI fibrosis score > 17.3.

**Table 4:** AI lamina propria fibrosis scores in patients with or without fibrosis-related endoscopic findings on index endoscopy (A), follow-up endoscopy, entire cohort (B), and follow-up endoscopy (subgroup of patients without strictures on index or prior endoscopies). Abbreviations: AI, artificial intelligence; LPF, lamina propria fibrosis; FSD, fibrostenotic disease; OP, original pathologist; CP, central pathologist; min, minimal; LP, lamina propria.

A. AI LPF scores—correlation with findings on index endoscopy							
		AI LPF score			AI LPF score		
	*N*=	Rings	No rings	*p*-value	Stricture	No stricture	*p*-value
All cases	180	19.3	8.0	*p* = 0.08	14.2	10.4	*p* = 0.50
Adequate, OP	93	23.6	16.2	*p* = 0.25	38.4	17.4	*p* = 0.14
Adequate, CP	91	44.0	20.7	*p* = 0.10	68.4	28.2	*p* = 0.20
Inadequate, OP+CP	60	6.5	3.9	*p* = 0.19	8.9	4.6	*p* = 0.56
Adults	92	19.3	5.6	**p** **=** **0.01**	15.0	10.5	*p* = 0.61
Pediatric	88	23.7	10.1	*p* = 0.12	13.2	10.4	*p* = 0.58
Diagnosed with EoE	166	19.3	11.0	*p* = 0.05	15.0	12.4	p = 0.58
Min. LP area of 0.05 mm^2^	144	23.8	14.4	**p** **=** **0. 009**	18.6	16.1	p = 0. 26
Min. LP area of 0.10 mm^2^	120	33.4	18.6	*p* = 0.06	20.8	23.6	*p* = 0.24
Min. LP area of 0.15 mm^2^	101	46.0	24.0	**p** **=** **0.03**	55.9	28.5	*p* = 0.17
Min. LP area of 0.20 mm^2^	84	54.8	23.0	**p** **=** **0.01**	68.4	32.5	*p* = 0.18


B. AI LPF scores—correlation with findings on follow-up endoscopy (entire cohort)										
		AI LPF score			AI LPF score			AI LPF score		
	*N*=	Rings	No rings	*p-*value	Stricture	No stricture	*p-*value	FSD requiring dilatation	FSD requiring dilatation	*p-*value
All cases	180	20.1	8.2	***p*** **=** **0.0005**	20.8	9.7	***p*** **=** **0.007**	20.8	9.7	**p** **=** **0.003**
Adequate, OP	93	46.5	13.0	***p*** **=** **0.0006**	48.7	17.3	p = 0.08	50.4	15.0	**p** **=** **0.03**
Adequate, CP	91	58.0	19.5	***p*** **=** **0.002**	93.8	25.6	***p*** **=** **0.04**	60.6	24.1	***p*** **=** **0.07**
Inadequate, OP+CP	60	8.2	3.8	**p** **=** **0.03**	14.2	3.9	***p*** **=** **0.005**	13.7	4.1	**p** **=** **0.02**
Adults	92	28.5	7.2	***p*** **=** **0.0004**	20.8	8.9	**p** **=** **0.02**	31.6	8.2	**p** **=** **0.008**
Pediatric	88	14.5	9.5	*p* = 0.26	22.7	9.8	p = 0.17	17.4	9.9	*p* = 0.22
Diagnosed with EoE	166	20.1	10.4	**p** **=** **0.005**	20.8	11.3	**p** **=** **0.02**	20.8	11.3	**p** **=** **0.01**
Min. LP area of 0.05 mm^2^	144	33.4	14.1	**p** **=** **0.003**	24.4	14.8	**p** **=** **0.04**	42.3	14.6	**p** **=** **0.01**
Min. LP area of 0.10 mm^2^	120	46.6	17.3	***p*** **=** **0.001**	47.4	20.4	**p** **=** **0.04**	49.1	20.4	p = 0.05
Min. LP area of 0.15 mm^2^	101	54.4	20.2	**p** **=** **0.0006**	55.0	26.1	**p** **=** **0.03**	55.0	26.1	**p** **=** **0.04**
Min. LP area of 0.20 mm^2^	84	55.9	20.7	**p** **=** **0.001**	65.3	28.8	**p** **=** **0.03**	55.5	28.5	p = 0.06


C. AI LPF scores—correlation with findings on follow-up endoscopy (patients w/o strictures)							
		AI LPF score			AI LPF score		
	*N*=	Stricture	No stricture	*p-*value	FSD requiring dilatation	No FSD requiring dilation	*p-*value
All cases	151	62.3	9.4	***p*** **=** **0.007**	45.8	9.5	***p*** **=** **0.01**
Adequate, OP	76	105.6	14.8	***p*** **=** **0.03**	71.2	14.8	p = 0.06
Adequate, CP	76	114.5	23.5	**p** **=** **0.02**	96.6	23.5	p = 0.07
Inadequate, OP+CP*	49	20.6	3.9	**p** **=** **0.04**	na	na	**na**
Adults	65	125.8	7.3	**p** **=** **0.01**	80.1	7.3	**p** **=** **0.03**
Pediatric	86	22.7	9.8	*p* = 0.16	17.4	9.9	p = 0.22
Diagnosed with EoE	137	62.3	11.0	**p** **=** **0.01**	45.8	11.1	**p** **=** **0.03**
Min. LP area of 0.05 mm^2^	119	62.3	14.5	**p** **=** **0.03**	45.8	14.5	p = 0.07
Min. LP area of 0.10 mm^2^	101	96.6	19.8	**p** **=** **0.03**	71.2	20.2	p = 0.06
Min. LP area of 0.15 mm^2^	83	114.5	24.7	**p** **=** **0.01**	105.6	25.9	**p** **=** **0.02**
Min. LP area of 0.20 mm^2^	67	125.8	28.2	**p** **=** **0.008**	105.6	28.2	**p** **=** **0.03**

P-valued <0.05 are shown in bold.

In the subgroup of patients without strictures on index or prior endoscopies, AI LPF score was also predictive of strictures on follow-up endoscopies in all cases and in all subgroups, except in pediatric patients (Table 4C). For the subgroup of patients without strictures, ROC analysis showed an AUC of 0.78 (*p* < 0.001) for prediction of strictures in this cohort at an AI fibrosis score > 9.9, AUC of 0.94 (*p* < 0.001) in the adult subgroup at score of >88.1, and an AUC of 0.71 (*p* = 0.003) in the pediatric subgroup at a cutoff of >9.9. A multiple regression model, which included AI fibrosis score, PEC (OP and CP), and total EoEHSS score (OP and CP), AI fibrosis score was the only statistically significant predictor of strictures on follow-up EGDs (*p* = 0.01) in patients without prior strictures.

The subgroup of samples containing any amount of LP (as assessed by the CP) but deemed inadequate for LPF evaluation by both the OP and CP (*n* = 41, 18 adults, 23 pediatric patients) revealed a median of 0.12 mm^2^ of LP (0.23 mm^2^ in adults and 0.09 mm^2^ in the pediatric population). In this subgroup, AI fibrosis scores correlated with the presence of rings on index EGD (20.8 vs 9.8, *p* = 0.004) and on subsequent EGDs (18.0 vs 9.7, *p* = 0.01). The AI fibrosis scores in these samples were also higher in patients with strictures on index and follow-up EGDs, but without reaching statistical significance.

In the subgroup deemed inadequate for LPF evaluation by both the OP and CP (*n* = 60), the AI model fibrosis score was predictive of the development of rings, strictures, and strictures requiring dilatation on subsequent EGDs. The model was also predictive of strictures in patients without history of strictures (Table 4A–C).

In the subgroup of patients with any amount of LP (as interpreted by the CP) and total LP area less than 0.32 mm^2^ (below the median amount required for adequacy by the CP), AI fibrosis score predicted rings (score of 10.4 vs 5.6 for patients with and without the outcome, respectively, *p* = 0.02) and strictures requiring dilatation (score of 15.8 and 6.0 for patients with and without the outcome, *p* = 0.03) on follow-up EGDs.

**Fig. 4:**
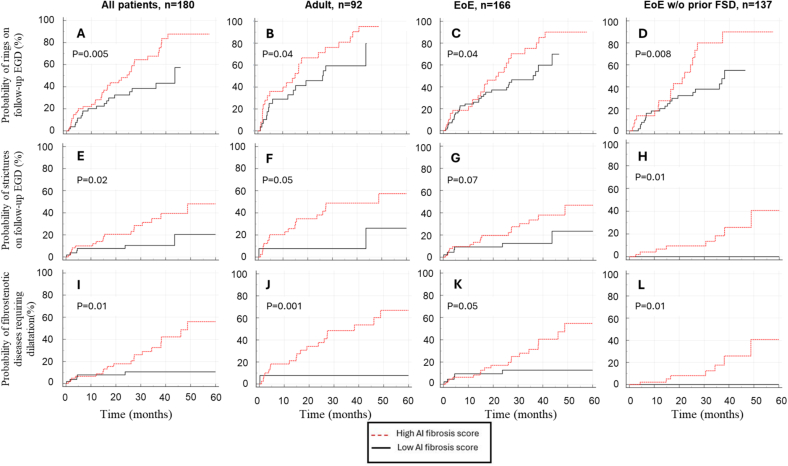
Kaplan–Meier analysis of AI model fibrosis score in the prediction of fibrostenotic disease. Probability of developing rings (A–D), strictures (E–H), and strictures requiring dilatation (I–L) on follow-up endoscopies. Abbreviations: EGD, esophagogastroduodenoscopy; EoE, eosinophilic esophagitis; FSD, fibrostenotic disease.

Kaplan–Meier analysis showed increased probability of fibrostenotic disease on follow-up endoscopy (with a median follow-up time of 31.4 months) in the high AI LPF score group (Fig. 4). A Cox proportional-hazards statistical model that included patient age, disease duration, and AI LPF score showed a C-index of 0.73 (0.64–0.82, *p* = 0.004) for prediction of strictures on subsequent EGDs, with LPF scores as an independent predictor. There was no correlation between patient age or disease duration with AI model LPF scores.

### Lamina propria adequacy by pathologists

According to the OPs, 49.7% (106/213) of all slides had an adequate amount of LP for fibrosis evaluation by subjective assessment, including 66.6% (66/99) of adults, and 35.0% (40/114) of pediatric patients. According to the CP, 47.4% (101/213) of all samples had an adequate amount of LP for fibrosis evaluation by subjective assessment, including 48.5% (48/99) of adult cases and 46.4% (53/114) of pediatric cases. Approximately two-thirds (63.9%) of samples were considered adequate by either the OP or CP. There was only fair agreement between OP and CP on LP adequacy (weighted Kappa of 0.38, 0.40, and 0.37 for all samples, adults, and pediatric subgroup, respectively) (Table 3).

The CP also evaluated the presence of any recognizable LP in the sample, regardless of whether this was deemed sufficient for LPF evaluation. LP was present in any recognizable amount in 66.6% of samples (142/213) but was classified as adequate in 47.4% of samples (101/213). Therefore, in nearly 20% of samples, there is a recognizable LP that is insufficient for analysis by routine pathological examination.

### Lamina propria measurement and adequacy by the AI model

The median area of LP present per histological level (3.4 levels were present on average per slide) was 0.18 mm^2^ overall, 0.21 mm^2^ in samples from the adults, and 0.14 mm^2^ in samples from the pediatric population, as measured by the AI model. This was due to the lower amount of total tissue present in the pediatric subgroup   compared to adults (3.31 mm^2^ vs 5.73 mm^2^, respectively, *p* < 0.001). Cases deemed adequate by the OP and CP contained a median of 0.26 mm^2^ and 0.32 mm^2^, respectively, of LP (per level) as measured by the AI model, compared to cases deemed inadequate, which contained 0.08 mm^2^ and 0.06 mm^2^, respectively.

LP area (unadjusted for total tissue area) was significantly higher in active disease (defined as ≥15 eosinophils/HPF by either the OP or CP) compared to inactive disease (0.04 vs 0.20 mm^2^, respectively, *p* = 0.0001). This difference was still significant (0.01 vs 0.05 mm^2^/mm^2^ of total tissue, *p* < 0.0001) when normalized for total tissue area. LP area (unadjusted for total tissue area) had a weak correlation with various other measurements of epithelial inflammation, including total EoEHSS by the OP and CP, PEC by the OP and CP, AI model PEC, AI model EoEHSS (*R*s of 0.38, 0.39, 0.35, 0.30, 0.34, and 0.28, respectively, (*p* < 0.0001 for all) as well as total EREFS (*R*_s_ of 0.21, *p* = 0.004). This correlation improved slightly after normalization for total tissue area, indicating that this association is not due to larger sample size (i.e., more extensive sampling during endoscopic examination) in cases with more disease activity. LP area correlated with LP fibrosis grade by pathologists (*R*s = 0.30), without significant change after normalization for total tissue area. LP area also correlated with LPF score by the AI model (*R*s = 0.70 for both the adult and pediatric subgroups, *p* < 0.0001), which did not change significantly after LP normalization for total tissue area (*R*s = 0.69).

Agreement between AI model adequacy cutoffs (minimum amount of LP in the sample) and pathologists' adequacy was highest at cutoff above 0.10 mm^2^ (Supplemental Table 6). Adopting a cutoff of 0.10 mm^2^ in our cohort would include just over 60% of the samples. ROC analysis between the AI-based LP area and pathologists' adequacy assessment showed an AUC of 0.73 (*p* < 0.001) at an optimum cutoff point of 0.11 mm^2^ (OP) and an AUC = 0.91 (*p* < 0.001) at an optimum cutoff point of 0.16 mm^2^ (CP).

## Discussion

LPF is a key histopathological feature in EoE and plays a central role in disease progression and long-term complications such as esophageal strictures, narrow esophagus, and episodes of food impaction.^1^, ^2^, ^3^^,^^15^ However, LPF histological assessment has been challenging due to technical difficulties in obtaining sufficient subepithelial tissue and in interpreting histological findings accurately and reproducibly. This is the first study, to our knowledge, which has leveraged highly accurate AI-based WSI analysis techniques to detect and quantify esophageal LP and LPF on biopsy samples in the context of EoE/mucosal eosinophilia evaluation utilizing routine hematoxylin and eosin stains. The accuracy achieved by our model has enabled us to investigate LP adequacy and fibrosis at a level of detail that has not been possible with traditional histopathological examination.

The main finding in our study was that the AI model LPF score correlated with pathologists' LPF assessment and was also predictive of fibrostenotic disease on follow-up endoscopies, including development of rings, strictures, and strictures requiring dilatation. This predictive ability was shown for the entire cohort and for most subgroups, including patients without a history of stricture on index or prior endoscopies. Importantly, the AI model LPF score also predicted fibrostenotic complications in the subgroup of samples classified as inadequate by both the OP and CP and in the subgroups of samples with measured LP below the average area in biopsies considered adequate by pathologists. For instance, whereas the median sample considered adequate by the CP contained 0.32 mm^2^ of LP, the AI model was predictive of fibrostenotic disease in the subgroup with LP area below 0.32 mm^2^ (median of 0.10 mm^2^), as measured digitally.

Previous studies have shown that only 42%–60% of esophageal endoscopic biopsy samples obtained from EoE patients contained an adequate amount of LP for fibrosis evaluation, as assessed by pathologists.^9^^,^^16^ Similarly, in our study, approximately half of the samples were considered adequate for LPF assessment by pathologists (slightly less than half in the pediatric population and slightly more than half in the adult population), but with only fair agreement for adequacy (Cohen's Kappa of 0.38) between the OP and CP. In prior studies, adequacy criteria for LPF evaluation have been based either on the pathologists' subjective impression of “sufficient” tissue for this interpretation,^11^ presence of any amount of LP,^9^ or minimal LP area based on the authors' experience.^7^ Our AI model data show that a minimum amount of LP (per paraffin block level) between 11 and 16 mm^2^ (OP and CP, respectively) represented the best cutoffs to distinguish adequate from inadequate biopsy samples, as interpreted by pathologists. However, our correlation data with fibrostenotic outcomes show that the AI model can detect meaningful predictive information in samples containing significantly less LP area than is typically required by pathologists. Therefore, our data indicate that establishing adequacy criteria based on the predictive ability of specific area cutoffs for fibrosis-related clinical outcomes would be a more useful approach.

All WSIs included in this study had some LP detected by the AI model, ranging from 0.003 mm^2^ (1.25% of a standard high-power field) to 2.8 mm^2^ (11.6 high-power fields), adjusted for number of histological sections. Whereas there must be a minimum LP area requirement for any given algorithm to have predictive ability, our data suggest that the adequacy requirement for LPF evaluation could be reduced at least from 0.26 mm^2^ (median requirement for the OP) to 0.08 mm^2^ (median LP area in the “inadequate” group by either OP or CP, for which the AI model LPF score was predictive of fibrostenotic complications). This reduction in the minimal LP area requirement resulted in the inclusion of approximately 30% of our samples, which otherwise would not have been evaluated for LPF [from 79/213 (37.0%), to 143/213 (67.1%)]. The reason pathologists tend to classify some cases that contain readily identifiable LP as “inadequate” is unclear. Histological review of this subgroup showed that these samples tended to have long, very narrow strips of LP along the base to the epithelium, rather than more localized areas of deeper LP seen in cases deemed adequate (Supplemental Fig. 1). However, the distribution of LP and its depth, which could potentially shed light on this issue, cannot be evaluated by our AI model with its current functionality.

In agreement with a prior study by Hiremath et al. analyzing the association between EoE activity and LP adequacy,^16^ our data showed that AI-based LP area measurement indeed correlated with disease activity. Hiremath and colleagues also questioned whether this was truly due to inflammation-related mucosal changes or simply to more extensive sampling in cases with more active disease, prompting endoscopists to take deeper and/or a larger number of biopsies. With precise LP measurements, we were able to demonstrate that the LP area did correlate (albeit weakly) with disease activity even after normalization for total biopsy area, arguing against the contention that more extensive sampling (rather than mucosal-specific characteristics or other factors) explains the larger amounts of LP tissue on histology slides. AI model LPF scores in our cohort had significantly higher correlation with LP area (with or without normalization for total tissue area) than did inflammatory features. However, the LPF score is calculated using the total fibrotic area (which is closely correlated with LP area – *R*s = 0.89). Therefore, our data cannot be used to test the hypothesis that LPF (rather than inflammation or other factors) is the primary driver of higher LP area in cases of active EoE, although this is a possible mechanism. Previous studies^17^, ^18^, ^19^ described the so called esophageal biopsy “pull sign”, or “tug sign”, whereby endoscopists feel resistance when pulling the forceps to obtain mucosal fragments in cases of EoE but not in non-EoE control subjects. The authors^17^ hypothesize that this endoscopic sign may be due to mucosal remodeling (which would include LPF, neovascularization, and possibly other features, but not intraepithelial inflammation), although no clear association with LPF was found in their study.

Our study also has limitations. First, although our model has been trained using WSIs obtained in several labs to maximize its generalizability, our validation cohort (for the prediction of fibrostenotic complications) was restricted to one reference center. This was due to the lack of consistent availability of standardized endoscopic reporting of fibrosis-related features (EREFS) in other participating institutions during the sample collection period. Therefore, the predictive ability of our model is yet to be demonstrated in external cohorts. Second, our study was underpowered to predict two low-frequency outcomes in our patient cohort: food impaction (an important complication in patients with EoE) and fibrostenotic complications in the pediatric subgroup. There was also significantly lower LP area in the pediatric vs adult population (likely unrelated to the use of pediatric forceps, which is used only in children up to ages 1–2 years at our institution, which represented less than 1% of our study cohort) and lower interobserver agreement for both LP adequacy and LPF evaluation in this population. It is also plausible that the degree of fibrosis involvement of deeper layers (i.e., submucosa and muscularis propria) in the pediatric population is not as extensive or established as in adults, leading to fewer outcomes despite the presence of similar rates and degree of fibrosis in superficial layers. Third, there were technical challenges in the recognition of LPF by our model, which included areas of forceps-related crush artifact and linear areas of relatively dense collagen immediately below the squamous epithelium (likely a normal finding), both of which were frequently erroneously recognized as fibrosis by our algorithm (i.e., false-positive segmentation). New versions of our model with improved exclusion of these problem areas are currently being developed by our group. Lastly, LPF is often accompanied by stromal hypercellularity (activated myofibroblasts), mixed inflammation, neovascularization, and smooth muscle hyperplasia. These features are now considered part of the mucosal “remodeling” process in EoE and may be relevant in the setting of LPF evaluation and prediction of fibrostenotic disease. Automated recognition of these features is under investigation by our group, but is not part of the model utilized in this study.

Despite these limitations, our AI model was able to analyze LP and LPF in esophageal biopsy samples with an unprecedented level of detail and, for the first time to our knowledge, provide LPF histological measurements that are predictive of fibrostenotic complications across several subgroups. In the current disease paradigm, EoE is viewed as a chronic inflammatory process which progresses to a predominantly fibrostenotic disease after a decade or more of ongoing injury. Therefore, a histology-based tool that can accurately measure LPF could provide valuable information about disease severity and probability of progression. This could be useful both for clinical and research purposes. Clinically, in an age when several therapeutic options are available, this tool could be used to identify patients at higher risk for fibrostenotic complications who would be candidates for treatment escalation. Patients with significant LPF scores could also be evaluated more closely with additional studies for fibrostenotic disease, such as barium swallow^20^ and EndoFLIP.^21^ Also, we have shown the potential for AI models to greatly increase the overall value of LP histological assessment, improving the utilization of the limited LP tissue present in esophageal samples obtained by current standard endoscopic techniques. More accurate, reproducible, and predictive LP histological analysis by AI may also increase gastroenterologists' interest in developing and utilizing biopsy techniques that can yield samples with larger amounts of LP,^22^ which could have greater predictive value when digitally analyzed. This could be particularly valuable in the pediatric population, in whom comparatively small LP areas may be negatively impacting LPF evaluation by both pathologists and machines. From a research perspective, a superior method of LP and LPF measurement could be valuable in further validating new LPF diagnostic modalities such as EndoFLIP,^23^^,^^24^ ultrasound-based techniques,^25^ and histology-based tools,^26^^,^^27^ and in evaluating treatment response to novel EoE therapies.

Our survey responses indicate that gastroenterologists would be likely to alter clinical management if provided with a validated, AI-derived fibrosis risk score. In particular, such a model could support more proactive surveillance strategies and therapeutic escalation by translating histological fibrosis into a reproducible and clinically interpretable risk metric. These findings suggest that AI-based quantification of LPF has the potential to enhance the clinical relevance of biopsy assessment by informing clinical decision-making beyond eosinophil counts alone. This interpretation is tempered by the relatively small sample size and the single-center nature of the survey, but nonetheless supports further development and validation of AI-driven fibrosis models in eosinophilic esophagitis.

Finally, beyond LPF, several additional biopsy-level features relevant to disease remodeling in EoE remain amenable to AI-based analysis. These include automated assessment of LP cellularity (e.g., activated myofibroblasts and mixed inflammatory infiltrates), neovascularization, smooth muscle hyperplasia, and spatial patterns of fibrosis distribution and depth, which may better reflect transmural involvement. Integration of fibrosis quantification with other AI-derived epithelial metrics (including dyskeratosis and surface epithelial change) may enable composite, multivariate risk models that more accurately predict disease trajectory and treatment response. In parallel, alternative methods for collagen assessment, including stain-free optical techniques such as second harmonic generation microscopy and other advanced imaging approaches, could provide complementary, collagen-specific information and may be well suited for AI-based interpretation alongside routine histology. Future work will also focus on external validation across institutions, longitudinal modeling of fibrosis evolution over time, and correlation with functional measures such as EndoFLIP and radiological assessments.

In conclusion, our work has shown that digital pathology and AI-based tools can add significant value to the histopathological analysis of LP and LPF in mucosal biopsies in the setting of EoE/esophageal mucosal eosinophilia. Our AI model was able to quantify LPF, generate scores that can predict fibrostenotic disease, and do so with significantly less LP tissue, including in the subset of cases deemed inadequate by pathologists.

## Declaration of competing interest

The authors declare that they have no known competing financial interests or personal relationships that could have appeared to influence the work reported in this article.
