# Supplementary material for: A machine learning model of lamina propria fibrosis in eosinophilic esophagitis for prediction of fibrostenotic disease
Source: J Pathol Inform. 2025 Dec 22;20:100538. doi: 10.1016/j.jpi.2025.100538 (PMC12828521; doi:10.1016/j.jpi.2025.100538)
Supplement: Supplementary material [file mmc1.docx]

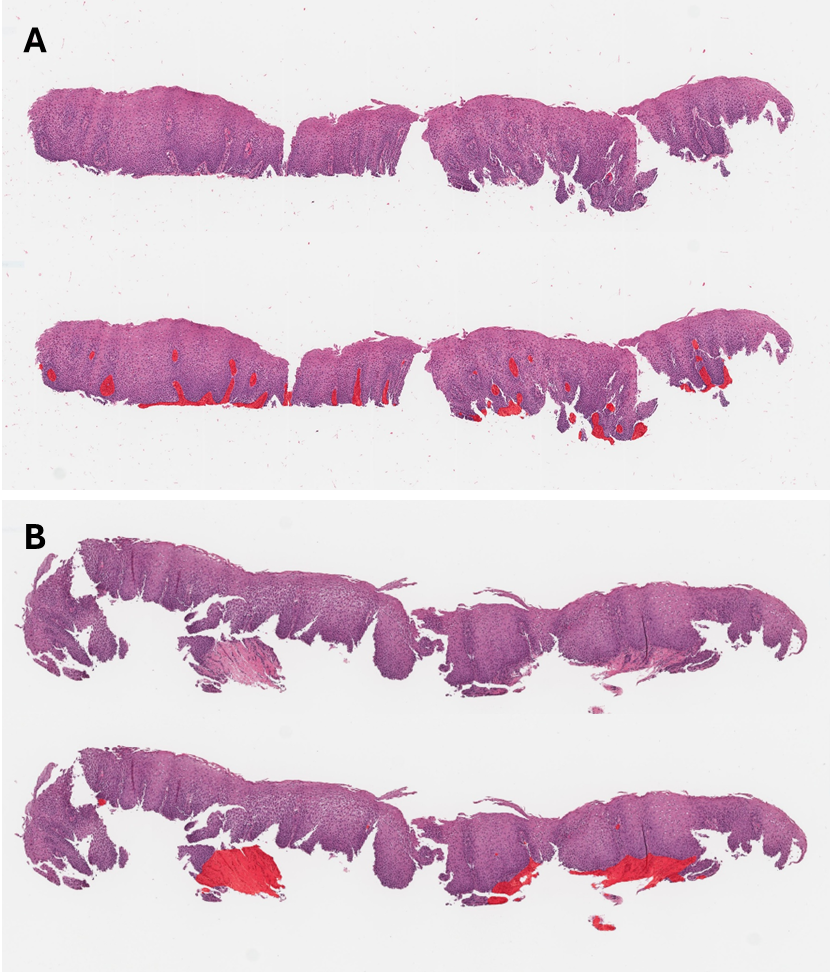


Supplemental figure 1. Lamina propria recognition by the artificial intelligence model (segmentation in red). Thin, superficial subepithelial strips of lamina propria, typical of cases deemed inadequate for lamina propria fibrosis evaluation by pathologists, A. Thicker, deeper chunks of lamina propria characteristic of cases considered adequate by pathologists, B.

|  | **Image Augmentation** | | | | | | | **Training Parameters** | | | | | |
| --- | --- | --- | --- | --- | --- | --- | --- | --- | --- | --- | --- | --- | --- |
|  | Scale (min/max) | Aspect Ratio | Max Shear | Luminance (min/max) | Contrast (min/max) | Max White Balance Change | Noise | Iterations without progress | Initial Learning Rate | Weight Decay | Neural Net Structure Version | Complexity | FOV |
| **Lamina propria fibrosis** | -1/1.01 | 1 | 1 | -1/1.01 | -1/1.01 | 1 | 0 | 1000 | 0.1 | 0.0001 | Default | Extra Complex | 25um |
| **Collagen and non-collagen** | -1/1.01 | 1 | 1 | -1/1.01 | -1/1.01 | 1 | 0 | 1000 | 0.1 | 0.0001 | Default | Extra Complex | 25um |

Supplemental table 1: Image augmentation and training hyperparameter details for model training within our artificical intelligence image platform (Aiforia Technologies). Abbreviations: min, minimal; max, maximal; FOV, field of view.

| **Full Question** | **Response Option** | **Frequency (n)** |
| --- | --- | --- |
| **1. How many years have you been in practice?** | <5 years | 7 |
|  | 5–10 years | 2 |
|  | 11–20 years | 2 |
|  | >20 years | 2 |
| **2. How many EoE patients do you personally manage per month (on average)?** | 0–5 | 5 |
|  | 6–15 | 5 |
|  | 16–30 | 1 |
|  | >30 | 2 |
| **3. Approximately how many endoscopic biopsy procedures for EoE or suspected EoE do you perform per month?** | Mean | 14.9 |
|  | Median | 15 |
|  | Range | 5–30 |
| **4. How strongly does lamina propria fibrosis influence your assessment of disease severity, beyond peak eosinophil count?** | Strong influence | 1 |
|  | Moderate influence | 4 |
|  | Weak influence | 8 |
|  | No influence | 0 |
| **5. How clinically useful do you consider lamina propria fibrosis information to be?** | Very useful | 2 |
|  | Moderately useful | 6 |
|  | Minimally useful | 4 |
|  | Not useful | 0 |
|  | Not sure | 1 |
| **6. In your experience, how well do you believe lamina propria fibrosis observed in biopsy correlates with endoscopic signs such as the “pull sign” or “tug sign”?** | Highly correlated | 2 |
|  | Moderately correlated | 6 |
|  | Minimally correlated | 4 |
|  | Not correlated | 1 |
|  | Unsure | 0 |
| **7. Based on your understanding of EoE-related fibrosis, does lamina propria fibrosis adequately reflect deeper wall involvement (e.g., muscularis propria fibrosis)?** | Yes | 2 |
|  | Possibly | 6 |
|  | Probably not | 4 |
|  | No | 1 |
|  | Unsure | 0 |
| **8. Based on your experience, is lamina propria fibrosis helpful in predicting future fibrostenotic complications?** | Yes, clearly | 1 |
|  | Yes, but needs more evidence | 7 |
|  | Uncertain | 4 |
|  | Probably not | 1 |
|  | No | 0 |
| **9. If a validated AI-based lamina propria fibrosis score predicted risk of future fibrostenotic complications, would you (select all that apply)?** | Escalate therapy | 8 |
|  | Increase monitoring frequency | 9 |
|  | Recommend specific testing modalities | 6 |
|  | Not change management | 1 |
| **10. What type of data would be most helpful for reporting fibrosis severity on histology?** | Numerical quantitative value | 5 |
|  | Categorical grading (mild/moderate/severe) | 6 |
|  | Risk stratification | 4 |
|  | Predictive score | 5 |
|  | Combination of the above | 7 |
|  |  |  |

Supplemental table 2. Survey among 13 of our gastroenterologists about their views on eosinophilic esophagitis-related lamina propria fibrosis and the potential relavance of an AI model in this context.

|  | **N** | **Rings, index EGD** | **Stricture, index EGD** | **Rings, FU EGD** | **Stricture, FU EGD** | **Dilatation, FU EGD** |
| --- | --- | --- | --- | --- | --- | --- |
| All | 180 |  |  |  |  |  |
| LPF (OP) | 63 | P=0.03 | NS | P=0.004 | NS | P=0.02 |
| LPF (CP) | 65 | P=0.02 | NS | NS | NS | NS |
| Adequate, OP or CP |  |  |  |  |  |  |
| LPF (OP) |  | NS | NS | P=0.003 | NS | P=0.03 |
| LPF (CP) |  | NS | NS | NS | NS | NS |
| No prior stricture | 151 |  |  |  |  |  |
| LPF (OP) |  |  |  | P-0.01 | NS | P=0.03 |
| LPF (CP) |  |  |  | NS | NS | P=0.04 |

Supplemental table 3. Presence of lamina propria fibrosis by original and central pathologists and correlation with fibrostenotic disease findings on endoscopy. “Index EGD” refers to the endoscopy procedure during which biopsies analyzed by the AI model were collected. Abbreviations: LPF, lamina propria fibrosis; OP, original pathologist; CP, central pathologist; FU, follow-up; EGD, esophagogastroduodenoscopy.

|  | **n (%)** | **AI fibrosis score** | **P value** |
| --- | --- | --- | --- |
| LPF absent, OP | 33 (15.4%) | 6.6 | Ref. |
| LPF present, OP | 73 (34.2%) | 34.1 | <0.0001 |
| Inadequate, OP | 107 (50.2%) | 6.0 | >0.05 |
| LPF absent, CP | 28 (13.1%) | 6.7 | Ref. |
| LPF present, CP | 74 (34.7%) | 47.9 | <0.0001 |
| Inadequate, CP | 111 (52.1%) | 5.6 | >0.05 |

Supplemental table 4. AI model fibrosis score in different subgroups. Abbreviations: LPF, lamina propria fibrosis; OP, original pathologist; CP, central pathologist.

| **Feature:** | **Correlation with AI LPF score:** | **P value** | **95% CI** |
| --- | --- | --- | --- |
| **Clinical** |  |  |  |
| Age | -0.01 | NS | -0.15–0.13 |
| Disease duration | -0.04 | NS | -0.26–0.18 |
|  |  |  |  |
| **Endoscopy:** |  |  |  |
| Total EREFS | 0.44 | <0.0001 | 0.32–0.61 |
| Edema | 0.25 | P=0.0009 | 0.11–0.39 |
| Rings | 0.23 | P=0.008 | 0.09–0.36 |
| Exudate | 0.39 | P=0.0001 | 0.26–0.51 |
| Furrows | 0.34 | P=0.0001 | 0.20–0.48 |
| Strictures | Categorical variable* | P=0.60 | — |
|  |  |  |  |
| **Routine histology:** |  |  |  |
| PEC (OP) | 0.57 | <0.0001 | 0.46–0.61 |
| PEC (CP) | 0.51 | <0.0001 | 0.41–0.61 |
| EI grade (OP) | 0.50 | <0.0001 | 0.39–0.61 |
| EI stage (OP) | 0.55 | <0.0001 | 0.45–0.62 |
| EI grade (CP) | 0.52 | <0.0001 | 0.41–0.61 |
| EI stage (CP) | 0.54 | <0.0001 | 0.44–0.62 |
| BZH grade (OP) | 0.53 | <0.0001 | 0.42–0.62 |
| BZH stage (OP) | 0.51 | <0.0001 | 0.41–0.60 |
| BZH grade (CP) | 0.58 | <0.0001 | 0.46–0.66 |
| BZH stage (CP) | 0.55 | <0.0001 | 0.44–0.64 |
| DIS grade (OP) | 0.52 | <0.0001 | 0.41–0.61 |
| DIS stage (OP) | 0.39 | <0.0001 | 0.22–0.53 |
| DIS grade (CP) | 0.44 | <0.0001 | 0.29–0.56 |
| DIS stage (CP) | 0.36 | <0.0001 | 0.16–0.54 |
| LPF grade+stage (OP) | 0.54 | <0.0001 | 0.43–0.64 |
| LPF grade+stage (CP) | 0.46 | <0.0001 | 0.28–0.55 |
| Total EoEHSS (OP) | 0.52 | <0.0001 | 0.41–0.61 |
| Total EoEHSS (CP) | 0.52 | <0.0001 | 0.40–0.61 |
|  |  |  |  |
| **AI model:** |  |  |  |
| PEC, grid method | 0.37 | <0.0001 | 0.24–0.51 |
| Avg EI/mm2 | 0.39 | <0.0001 | 0.27–0.52 |
| Avg lymphocytes/mm2 | 0.31 | <0.0001 | 0.17–0.44 |
| BZH% | 0.34 | <0.0001 | 0.21–0.48 |
| DIS% | 0.14 | 0.03 | -0.01–0.28 |

Supplemental table 5. Correlation between main clinical, endoscopic, histologic, and AI model-based features with AI LPF scores. All correlations are calculated using Spearman’s rank correlation, except for EREFS strictures (categorical variable) for which Mann-Whitney test for independent samples was used. Abbreviations: AI, artificial intelligence; LPF, lamina propria fibrosis; EREFS, Edema, Rings, Exudate, Furrows, and stricture; PEC, peak eosinophil count; OP, original pathologist; CP, central pathologist; EI, eosinophilic inflammation; BZH, basal zone hyperplasia; DIS, dilated intercellular spaces; EoEHSS, eosinophilic esophagitis histologic scoring system; Avg, average; mm, millimeter.

| AI model criterion: | | Agreement on adequacy (Cohen’s Kappa) | | | | | |
| --- | --- | --- | --- | --- | --- | --- | --- |
|  |  | Original Pathologist | | | Central Pathologist | | |
|  | n (%) | All | Adult | Pediatric | All | Adult | Pediatric |
| >0.05 mm^2^ | 166 (77.9%) | 0.23 (0.10–0.36) | 0.34 (0.13–0.55) | 0.18 (0.03–0.34) | 0.39 (0.27–0.51) | 0.46 (0.29–0.64) | 0.33 (0.16–0.49) |
| >0.10 mm^2^ | 135 (63.3%) | 0.37 (0.25–0.50) | 0.38 (0.18–0.58) | 0.33 (0.17–0.50) | 0.61 (0.51–0.72) | 0.58 (0.42–0.74) | 0.64 (0.50–0.78) |
| >0.15 mm^2^ | 115 (53.9%) | 0.35 (0.23–0.48) | 0.29 (0.09–0.49) | 0.36 (0.19–0.54) | 0.64 (0.54–0.75) | 0.54 (0.37–0.70) | 0.74 (0.61–0.86) |
| >0.20 mm^2^ | 98 (46.0%) | 0.36 (0.24–0.49) | 0.30 (0.11–0.49) | 0.37 (0.19–0.55) | 0.58 (0.47–0.69) | 0.48 (0.30–0.65) | 0.66 (0.52–0.80) |

Supplemental table 6. Agreement on lamina propria adequacy between the AI model (at different cutoffs for adequacy) and pathologists, using Cohen’s Kappa. AI model adequacy cutoffs are adjusted for number of tissue levels present per whole slide image. Abbreviations: AI, artificial intelligence; n, number of cases; mm, millimeter.
